# Habitat stability, predation risk and ‘memory syndromes’

**DOI:** 10.1038/srep10538

**Published:** 2015-05-27

**Authors:** S. Dalesman, A. Rendle, S.R.X. Dall

**Affiliations:** 1 Institute of Biological, Environmental and Rural Sciences, Aberystwyth University, Aberystwyth, U.K; 2 Biosciences, College of Life and Environmental Sciences, University of Exeter, Exeter, U.K; 3 Centre for Ecology and Conservation, Biosciences, College of Life and Environmental Sciences, University of Exeter, Penryn, U.K

## Abstract

Habitat stability and predation pressure are thought to be major drivers in the evolutionary maintenance of behavioural syndromes, with trait covariance only occurring within specific habitats. However, animals also exhibit behavioural plasticity, often through memory formation. Memory formation across traits may be linked, with covariance in memory traits (memory syndromes) selected under particular environmental conditions. This study tests whether the pond snail, *Lymnaea stagnalis*, demonstrates consistency among memory traits (‘memory syndrome’) related to threat avoidance and foraging. We used eight populations originating from three different habitat types: i) laboratory populations (stable habitat, predator-free); ii) river populations (fairly stable habitat, fish predation); and iii) ditch populations (unstable habitat, invertebrate predation). At a population level, there was a negative relationship between memories related to threat avoidance and food selectivity, but no consistency within habitat type. At an individual level, covariance between memory traits was dependent on habitat. Laboratory populations showed no covariance among memory traits, whereas river populations showed a positive correlation between food memories, and ditch populations demonstrated a negative relationship between threat memory and food memories. Therefore, selection pressures among habitats appear to act independently on memory trait covariation at an individual level and the average response within a population.

Predation pressure exerts a significant selective pressure on behaviour, both in terms of evading predators, but also avoiding unnecessary antipredator responses that may reduce time available for foraging and reproduction^1^. In habitats where predation pressure is stable, local adaptation to predation environments may occur where innate responses to cues from a predator are enhanced in populations that overlap in distribution with that predator^2^,^3^,^4^,^5^. Predation pressure also exerts selection on a range of other traits within individuals, with populations from low-risk environments typically demonstrating increased boldness and activity levels reflecting lack of risk in their environment^6^,^7^,^8^. Predators may also exhibit strong indirect effects on prey behaviour^9^. One factor that has received considerable attention is how predators influence the foraging behaviour of their prey in tri-trophic systems, with ecological interactions among species occupying three trophic levels, predators, prey (a herbivore) and plants^10^,^11^. Foraging behaviour is often indirectly affected by predation risk via trait mediated indirect interactions (TMIIs), such that the foraging behaviour of a herbivore, for example, alters due to the presence of a predator. Therefore the predator may indirectly impact on plant growth in the habitat. Prey may choose to forage in less risky habitats or during different time periods when faced with predation threat^12^,^13^, and prey often become less selective about food resources in the presence of predation threat^14^.

Selection on plasticity in foraging and antipredator traits may act in two different ways. Firstly, it may act on the overall trait plasticity, i.e. how plastic an animal is in response to food resources or the predation environment. Secondly, plasticity in behavioural traits may be linked via covariation in memory formation across traits (i.e. memory syndromes), where the degree of plasticity an animal exhibits in response to its environment co-varies across different types of behaviour (e.g. Fig. 1a). Memory formation may also differ across behavioural traits, for example only altering a single behavioural trait but still maintaining covariance between behavioural traits in individuals (Fig. 1b). Alternatively, memory formation that differs either in the degree to which it alters behavioural traits (Fig. 1c) or among individuals within a population as well as across different traits (Fig. 1d) could either break down or enhance covariance among behavioural traits altering behavioural syndromes. For example, in wild *Gasterosteus aculeatus* (three-spine stickleback), high predation risk selects for correlations among suites of behaviours related to exploratory and risk-related behaviours; whereas low-risk populations demonstrate a lack of correlation among these traits^7^,^8^,^15^,^16^. However, recent exposure to a novel predation threat has been shown to both enhance^17^ and break down^18^ correlations among behavioural traits in *G. aculeatus*. Therefore, whilst some traits may be fixed, plasticity in traits may also form an important element of behavioural syndromes.

Habitat stability is predicted to exert differing selection pressures on behavioural flexibility among populations^19^. Plasticity in behavioural traits can occur though memory formation, allowing animals to react to their environment. Whilst memory ability is frequently assessed as a single trait in the context of behavioural syndromes, or the effect of experience is measured on a suite of unrelated ‘personality’ traits, co-variation in memory forming ability across different traits (a memory syndrome) has yet to be demonstrated in wild populations^20^,^21^,^22^. Memory traits can be defined by the ability of an animal to demonstrate flexibility in a behaviour following experience based on learned responses in different behavioural contexts rather than as a result of other physiological or morphological changes that may take place. Similarly to other traits an animal possesses, we might predict that memory will also differ in a consistent manner among individuals. A strong linkage between different memory traits would be predicted where a balanced response in both traits carries a greater fitness value than responding to each independently, whereas memory forming ability across traits may become disassociated if there is no fitness value to this linkage.

To test whether habitat type affects memory syndromes across different traits, we used the great pond snail, *Lymnaea stagnalis*. This species has two distinct advantages. Firstly, there are a number of well-defined memory traits that have been assessed using this species in the context of neurobiology and ecology^23^,^24^. Secondly, we have access to multiple populations and laboratory strains that come from different backgrounds of habitat stability and predation pressure. River populations experience a relatively stable habitat with predatory fish, whereas ditch populations come from relatively unstable habitats experiencing predation threat from a wide range of invertebrate predators. These factors may act independently in their selective pressure on memory formation; however, it is also possible that they will interact to affect memory. As these factors co-vary within the habitats from which wild populations were sourced, the current study does not attempt to isolate individual effects. These wild populations do exhibit innate differences in antipredator behaviour relative to the predator regime they experience, indicating that predation pressure has a significant effect on behavioural traits in this species^4^. Populations from each habitat type were bred through to the F1 generation using wild-caught adults (minimum of 50 to establish laboratory populations), and the F1 adults were used to assess memory traits. Laboratory strains have also been established for studies in genetics and neurobiology, allowing access to populations that have lived in very stable predation-free environments over many (≥14) generations. Adults from each habitat type were tested for long-term memory formation in three traits, two food-related (food aversion and food appetitive conditioning) and operant conditioning of aerial respiration. It has been proposed that operant conditioning is a threat aversion behaviour, related to antipredator behaviour^25^. Whilst adults do not demonstrate overt antipredator responses^26^, juveniles of this species do, and have been shown to form associative memory of predation threat^24^. To confirm if there is population level co-variance in juvenile antipredator behaviour and operant conditioning, we also assessed memory of predation threat in F2 juveniles from the river populations.

Memory formation across traits in *L. stagnalis* was therefore evaluated by: i) testing whether the average memory forming ability across the different traits in adults is consistent within habitat types; ii) determining whether memory of operant conditioning in adults was linked with memory of predation threat in juveniles at the population level; iii) assessing whether memory formation across adult memory traits covaries at an individual level (i.e. a ‘memory syndrome’); and iv) determining if the strength of covariation in memory formation is affected by habitat of origin. We predicted that in snails originating from habitats where relatively stable ecological problems (i.e. foraging and predation risk) co-occur, there would be stronger selection for memory syndromes (covariation among memory traits). In a relatively stable environment, retaining information about past experience is predicted to de-value at a slow rate as memory of recent experiences maintain a benefit for longer. Therefore river snails were predicted *a priori* to demonstrate better memory retention across all traits. Consequently, we also expected to find the strongest memory syndromes in river populations, i.e. where memory formation across traits shows strong positive covariation, and conversely we predicted little covariation among memory traits in the unstable ditch populations. In contrast, as potential to form memory carries costs in other species^27^, we predicted that laboratory populations that have been under relaxed selection for multiple generations would demonstrate poorer memory forming capabilities relative to river populations. Additionally, if selection in wild populations is maintaining co-variation among traits, this would also be lost in laboratory populations.

## Results

### Adult memory among populations

Adult memory in F1 snails from 8 populations (2 laboratory; 2 ditch; and 4 river) was tested using three traits, operant conditioning of aerial respiration (decrease in breathing behaviour = memory), aversive food conditioning (decrease in bite rate = memory) and appetitive food conditioning (increase in bite rate = memory). Each individual received all three training regimes one week apart over three weeks. Controls, where snails received the same number of stimuli but non-contingently, were used to determine memory formation. The estimated difference (including 95% confidence intervals) in response between contingent vs. non-contingent training and effect size for each population are given in Table 1.

**Figure 2:**
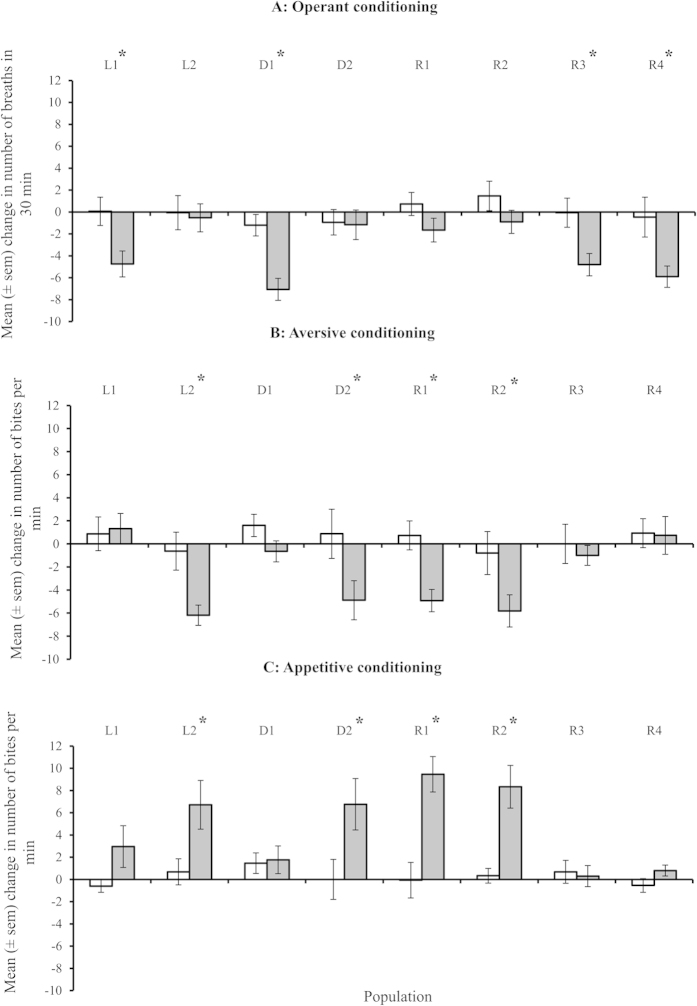
Population level memory response in adult snails across three traits. Populations derived from laboratory reared stock (L1-L2), ditch habitats (D1-D2) or river habitats (R1-R4). Mean change in behaviour (operant conditioning: breathing rate; aversive conditioning: bite rate; appetitive conditioning: bite rate) following non-contingent (white columns) or contingent (grey columns) training. * = significant effect of training (contingent vs. non-contingent) on the response (Student-Newman-Keuls pair-wise comparisons: P < 0.05). (N = 15-23 per treatment group).

Operant conditioning: the response to training differed among populations (Fig. 2; 2-way interaction: training regime*population(origin): F_5,15.11_ = 3.55, P = 0.026; η^2^_p_ = 0.540). Half of the populations tested demonstrated a significant decrease in breathing attempts 24 h following contingent training compared to those receiving non-contingent training (Fig. 2; Table 1), indicating that these populations had formed long-term memory. The order in which adult snails received training did not affect memory formation. There was also no significant effect of habitat of origin on memory formation.

Aversive conditioning: there was a significant response to training regime during aversive conditioning, with only contingently trained snails demonstrating a significant reduction in bite rate (Fig. 2; main effect of training: F_1,5.05_ = 8.01, P = 0.036; η^2^_p_ = 0.613; difference between control vs. trained = -3.100, CI: -1.647,-4.553). There was also a non-significant trend towards an effect of population on how snails responded to training (2-way interaction: training regime*population(origin): F_5,15.11_ = 2.75, P = 0.059; η^2^_p_ = 0.476), which is substantiated by a significant difference between non-contingently and contingently trained animals in half of the populations tested (Fig. 2; Table 1). The order in which adult snails received training did not affect memory formation. There was also no significant effect of habitat of origin on memory formation.

Appetitive conditioning: the response to training differed among populations (Fig. 2; 2-way interaction: training regime*population(origin): F_5,15.05_ = 4.75, P = 0.008; η^2^_p_ = 0.612). Half of the populations tested demonstrated a significant increase in bite rate in response to amyl acetate exposure 24 h following contingent training compared to those receiving non-contingent training, indicating that these populations had formed long-term memory (Fig.2; Table 1). The order in which adult snails received training did not affect memory formation. There was also no significant effect of origin on memory formation.

Overall, there was a pattern for population variability in long-term memory formation across the three traits. Populations that demonstrated good long-term memory following operant conditioning were poor at forming food related memories and vice versa (Fig. 2; Table 1). Habitat type populations originated from did not appear to affect which memories the snails are good at forming.

### Juvenile memory

Juvenile memory of a predation event was assessed in F2 individuals from the four river populations used to test adult memory. Juvenile snails were pre-exposed to predation or control cues and then tested using either predator kairomones or control pond water to determine if their antipredator behaviour (crawling out of the water) increased indicating memory of recent predation threat. The data were analysed including the phenotype of the F1 generation derived from adult memory traits: phenotype A came from populations where adults demonstrate good food memories, but poor operant conditioning memory, phenotype B came from populations exhibiting poor food memories, but good operant conditioning memory.

**Figure 3:**
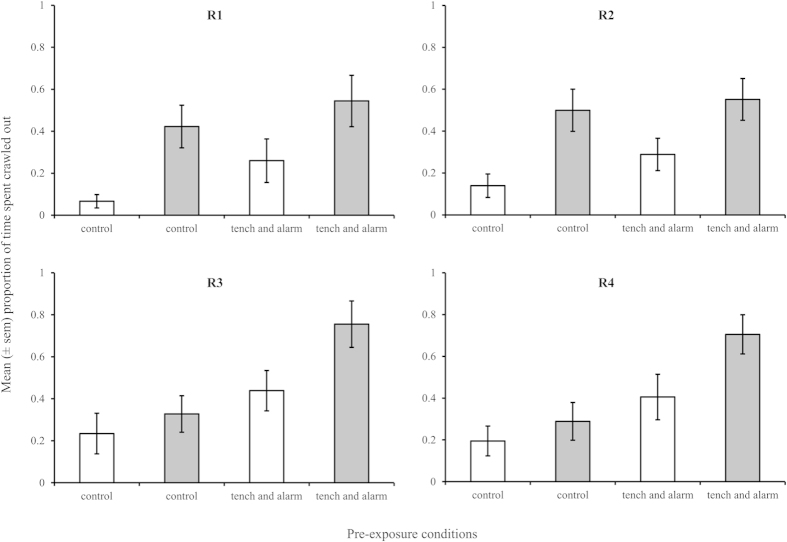
Antipredator behaviour of juvenile snails from four river populations (R1-R4) following cue association. Mean proportion of time spent crawled above the waterline in response to control pond water (white columns) and tench cue (grey columns) following pre-exposure to pond water alone (control) or tench and alarm cues. (N = 15 per treatment group).

Crawl out behaviour differed between the two phenotypes dependant on both pre-exposure conditions and exposure during the behavioural trial (Fig. 3; 3-way interaction: phenotype*pre-exposure*behavioural trial exposure: F_1,2_ = 66.63, P = 0.015; η^2^_p_ = 0.972 ). Phenotype A snails (from populations that demonstrate poor operant conditioning memory) demonstrated an elevated crawl out response to tench cue during behavioural trials relative to snails that had received control conditions throughout (SNK: P < 0.05; difference 0.441, CI: 0.255,0.627; η^2^_p_ = 0.164), though pre-exposure did not significantly increase the crawl out response to tench cue alone (SNK: P > 0.05). For phenotype B snails (from populations that demonstrate good operant conditioning memory) there was no significant difference among groups pre-exposed to control conditions (irrespective of behavioural trial conditions) and those pre-exposed to tench plus alarm but exposed to control cues during the behavioural trial (SNK: P > 0.05). However, phenotype B snails pre-exposed to tench plus alarm cues then exposed to tench cues during the behavioural trial demonstrated a significantly elevated crawl out response to tench cues relative to phenotype B snails pre-exposed to control conditions (SNK: P < 0.05; difference 0.643, CI: 0.362,0.923; η^2^_p_ = 0.273). This indicates that the phenotype B snails have retained information about predation threat from their experience 24 hours previously and this memory of a recent predation event has elevated their response to the predator cues. There was no significant effect of population nested within phenotype on crawl out behaviour.

Phenotype A snails failed to demonstrate associative conditioning of predation threat 24 h following exposure, whereas phenotype B snails demonstrated a significantly elevated crawl out behaviour to tench cues following cue association learning. This indicates that they adjusted their antipredator behaviour based on recent experience as found in previous work^24^. Therefore, we concluded that operant conditioning in adults can be used as a proxy for memory about predation threat at a population level, as postulated in our previous study^25^.

### Memory syndromes

The data from memory formation in adult snails was also assessed at an individual level to determine if ability to form memory co-varied across the different adult memory traits, i.e. a ‘memory syndrome’, using their responses to operant conditioning, aversive conditioning and appetitive conditioning. All data were converted such that a positive value in the trait would be an indicator of good memory formation; therefore a positive correlation means that individuals that were good at memory formation in one trait were also good at memory formation in the other.

Following non-contingent training snails did not show any consistency in how they altered their behaviour between training and testing. However, following contingent training there was a significant relationship between how well snails formed memory in each memory trait. This relationship was negative between memory formation in operant conditioning and the two food related traits (operant vs. aversive: r = -0.23 (CI -0.379,-0.069), P = 0.007; operant vs. appetitive: r = -0.21 (CI: -0.361,-0.048), P = 0.012: N = 143), but there was a positive relationship between the two food memory traits (r = 0.22 (CI 0.058,0.37), P = 0.008, N = 143).

**Figure 4:**
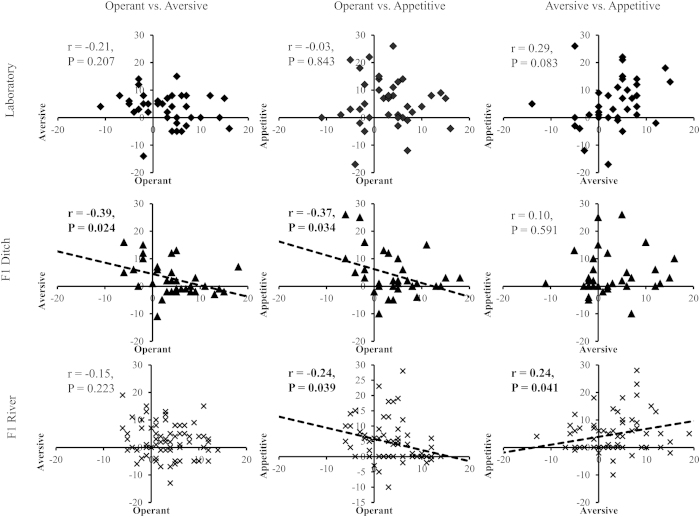
Correlation for individual memory formation among memory traits in snails derived from three habitat types. Positive value on x- or y-axis shows strength of memory formation (higher positive value = stronger memory in the trait). Trend line is included where Pearson’s correlations were significant (P < 0.05).

When data from each habitat type (laboratory, ditch and river) were analysed separately, there were differences in consistency in memory formation across traits compared to the overall pattern. Again, non-contingently trained individuals did not demonstrate consistency in how they altered behaviour between training and testing, indicating that without memory formation there is no evidence of behavioural syndromes across traits. However, following contingent training, habitat of origin affected the level of consistency among memory traits. Laboratory reared snails demonstrated no strong link among traits (Fig. 4; N = 38). Ditch origin snails showed a negative correlation between their ability to form food-related memories and their ability to form memory of operant conditioning (operant vs. aversive: r = -0.39 (CI: -0.058, -0.648), P = 0.024; operant vs. appetitive: r = -0.37 (CI: -0.032,-0.633), P = 0.034; N = 33), but no individual consistency in response across the two food-related memory traits (Fig. 4). Whereas river populations demonstrated a positive correlation between their ability to form memory in the two food-related memories (r = 0.24 (CI: 0.01,0.447), P = 0.041), and a negative association between appetitive conditioning and operant conditioning (r = -0.24 (CI: -0.45,-0.014), P = 0.039), but no consistency in response between operant and aversive conditioning (Fig. 4; N = 72). Overall these data show that consistency in how well individual snails perform across different memory traits is linked to the habitat they originate from, demonstrating habitat specific memory syndromes irrespective of the mean population response to training.

## Discussion

This study demonstrated that memory formation across four fitness-related traits differs significantly among *Lymnaea stagnalis* populations. Populations that exhibited strong memory in threat avoidance traits (predator cue association and operant conditioning) exhibited poor memory in foraging-related traits (food aversive and appetitive conditioning). Conversely, those that exhibited good food memories were inflexible in their threat avoidance behaviour. These population-level responses were not habitat specific, as might be predicted based on work with other species differing in predator regime^28^,^29^, but were distributed equally across different habitat types for the eight populations tested. If memory formation carries significant costs^27^, removing the benefits of memory under the relaxed selection conditions in the laboratory might be predicted to result in poorer memory formation in these individuals. A lack of effect of habitat of origin indicates these laboratory populations do not differ significantly in their ability to form memory relative to their wild counterparts. This suggests that either there are low costs associated with memory potential for these traits, or that the conditions in the laboratory, with food provided *ad libitum,* easy mating opportunities, little need to move far and a lack of predators, negate the costs associated with memory potential.

Why populations differ in their ability to form memory across the different traits is still to be determined. It could be that differences in physiology, including metabolic rate, alters whether animals are able to demonstrate plasticity. For example, metabolic rate may determine the scope an animal has to alter its feeding behaviour or the time it is able to allocate to threat avoidance. Differences in memory formation may also result from attentional bias rather than underlying differences in physiology or neural capability to form memory *per se*^30^. How individuals respond to stress for example, is highly likely to alter their memory retention^31^,^32^, and may affect the way an individual behaves in the novel environment used to train the snails. There is a strong correlation between the neurophysiological changes that take place in *L. stagnalis* and the change in behavioural phenotype following memory formation in both operant^33^ and appetitive conditioning^34^. This indicates that differences in how individuals respond to training are not due to behavioural masking of memory formation, but are instead due to underlying differences in the ability of the animals to form memory across the different traits. There is also evidence that neurophysiological differences among populations may determine how well the snail forms memory in response to operant conditioning at least^35^, indicating that underlying differences among individuals in their physiology drives the population variability we see in memory formation.

The ability of animals to perform consistently across a range of contexts, termed animal personality when assessed by the same trait over time or behavioural syndrome when assessed across different traits, has received significant interest in recent years^22^,^36^, particularly the role that this co-variation may play in population ecology^37^. How an animal responds to its environment can also be plastic, and the ability to learn and remember experiences can play an important role in this plasticity^21^. So far, evidence for individual consistency in memory formation across different traits among natural populations has proved elusive^38^. However, in *L. stagnalis*, we found evidence that covariation among memory traits - memory syndromes - do exist in wild populations. Individual consistency was identified across populations in the negative relationship between memory of threat avoidance and memory in food-related traits, which reflected the population level relationships among traits. Similarly, a positive relationship between the two food-related traits was also found. The effect sizes of these relationships were relatively low (r = 0.21-0.23), though within the normal range of individual levels of consistency in behaviour across many studies of behavioural syndromes^39^. However, when individual responses were assessed within habitat type, a different pattern becomes evident, demonstrating an effect of habitat in the strength of trait covariance as we would have expected *a priori* (see introduction). Nevertheless, the observed pattern did not conform to our habitat specific predictions for wild populations, and was considerably more complex than expected.

Pace-of-life syndromes, where individuals within populations differ in behavioural tendencies depending on metabolic and life-history requirements^36^, may explain why the strength of correlation among memory traits differs among habitat types in the opposite direction to our initial prediction. In unstable habitats with fluctuating predation threat, where refuge use becomes unreliable due to a diverse range of predator foraging activities, there is likely to be strong selection on life-history traits that allow survival in the face of continuous and variable threat. Unpredictable conditions may strongly favour individuals exhibiting alternative memory phenotypes, benefitting either fast growth rate and high reproductive output or long-lived threat aversive individuals. The relatively strong negative relationship (effect size r = -0.37 to -0.39) between threat aversion and food memories supports this hypothesis. As an individual, it is beneficial in ditch habitats to either demonstrate plasticity in response to foraging related cues, allowing fast growth and earlier reproductive output, or respond to predation threat, increasing longevity. Individuals that demonstrate a middle ground, between these two life-history strategies, may be disadvantaged.

In stable habitats, individuals may exhibit some degree of innate recognition of resources or predation threat. For example, there is strong evidence for innate predator recognition by *L. stagnalis* in river habitats found here and elsewhere^4^. Whilst some populations are clearly capable of altering their response following experience of predation cues^24^, those that do not are still afforded some degree of protection through this innate antipredator behaviour. Where predators are easily avoided through refuge use, selection on plasticity of avoidance mechanisms may be relatively weak if animals are able to demonstrate adaptation of innate responses. Instead, selection may act primarily on foraging behaviour, where animals are able to make use of food patches in safe places and can demonstrate a greater degree of selectivity based on food quality in stable habitats. Selection on pace-of-life phenotype may therefore be relaxed to some degree. In river populations, there is a positive relationship in food memory formation across the two traits, and also a negative relationship between threat aversion and food appetitive conditioning with similar effect sizes (r = 0.24) to the combined data, but the strength of these relationships is lower than that found in the ditch populations.

In laboratory populations, despite population level consistency in how well snails formed memory across the traits, there was little evidence of individual consistency in memory formation. A non-significant trend (P = 0.089) with a relatively strong effect size (r = 0.29) was found between the two food memory traits, indicating that laboratory rearing had not completely eliminated this linkage. However, there was no relationship between threat aversion and food traits. In the absence of predation threat (other than scientists) and a constant food supply, there is no selective benefit derived from memory formation across these traits. Whilst strain differences have been maintained over many generations in the laboratory environment, individual consistency in the relationship among memory traits appears to have been lost. This is unlikely to be a result of rearing conditions only, as all populations tested were F1 laboratory reared, but more likely a result of relaxed selection for this linkage between traits^40^. Together these data suggest that selection pressures within each habitat type are acting differently on links between memory traits, mirroring environmental effects on behavioural syndromes among populations^16^.

Memory syndromes may link in with the overall behavioural syndrome, not just in terms of how memory alters behavioural traits, but also how other behavioural traits may predict memory formation across different contexts. For example, a timid individual may form better threat aversion memories but poor food memories in a novel context where fear is elevated; a bold individual may be equally capable of forming food and threat related memories in the same novel context. However, in safer, familiar surroundings, both individuals may perform equally well. Memory syndromes are therefore likely to play a key role in understanding the evolutionary and ecological relevance of behavioural syndromes in wild populations^21^. Together these data point towards the importance that ecological background can play in determining the strength of covariation among traits underpinning behaviour^41^, whilst not having any apparent effect on the mean population behavioural responses.

## Methods

### Animal origin and maintenance

Pond snails, *Lymnaea stagnalis*, were used from eight original different sources. Two strains originated from laboratory populations that had been maintained under constant conditions in the laboratory for a minimum of 14 generations (L1-L2). Four strains were F1 laboratory reared adults originating from adults collected from river populations (R1-R4), and two strains were F1 laboratory reared adults originating from adults collected from ditch populations (D1-D2). Both river and ditch populations were collected on the Somerset Levels, U.K using sweep netting in aquatic vegetation, with a minimum of 50 adults collected per population and contributing to each generation. *Lymnaea stagnalis* is a preferentially out-crossing hermaphrodite mating frequently in the laboratory^42^, ensuring the maintenance of genetic variation in the laboratory populations. River populations are exposed to high levels of fish predation, with *Tinca tinca* (tench), a specialist molluscivore present at all sites. Ditch sites have no predatory fish present but experience invertebrate predation from bugs, leeches and beetles. Juveniles from ditch and river sites have been found to differ in their innate response to fish predation threat in previous work^4^. The ditch sites are also subject to frequent infilling from rotting vegetation, followed by dredging by farmers, so fluctuate in terms of vegetation available for food, water depth and oxygen availability (particularly during shallow, in-filled periods) to a greater extent than river populations^25^.

Adult snails (spire height 25 ± 1 mm) used for all experiments were reared under constant conditions in the Aquatic Resource Centre at the University of Exeter. They were held at 20 ± 1 °C on a 14:10 light:dark schedule in aerated artificial pond water (Ca^2+^ [80 mg/l]; Mg^2+^ [4.9 mg/l]; NaHC0^3^ [3.75 mg/L]; KCL [1.0 mg/L]; Marine salts (Crystal Sea® M*arinemix*, Baltimore, U.S.A) [20 mg/L]) and fed lettuce and trout pellets ad libitum. F2 juveniles (spire height 6 ± 0.5 mm) were reared under identical conditions to the adults.

### Training – adult memory

Adults were trained using three different methods: operant conditioning of aerial respiration^43^, food aversion conditioning^44^, and food appetitive conditioning^45^. Individuals from each population were randomly allocated to the contingent (trained) or non-contingent (control) group (see below for details). If changes in behaviour were due to memory formation, it was predicted that only trained snails that had received contingent stimuli would demonstrate a significant change in behaviour. Individual snails were exposed to all three training methods, randomly assigned to one of four orders in which they received each training method (contingently trained or non-contingent control). The four possible orders in which they received training were: 1) operant > aversive > appetitive, 2) operant > appetitive > aversive, 3) aversive > appetitive > operant and 4) appetitive > aversive > operant. The order in which they receive the different training methods was included in the subsequent analyses to assess whether forming memory under one regime altered memory formation under other regimes.

### Operant Conditioning

Snails are trained to associate a spontaneous behaviour (aerial respiration in hypoxic conditions) with a negative tactile stimulus. Memory is demonstrated by a reduction in breathing behaviour in hypoxia in trained animals but not in non-contingent controls.

Contingent (trained): 500 ml of artificial pond water was placed in a 1 l glass beaker. N_2_ was then vigorously bubbled through the water for 20 min to make the water hypoxic (<5% [O_2_]). N_2_ bubbling was reduced and continued at a low level to maintain hypoxic conditions without disturbing the animals. Snails were then introduced into the beaker in small groups of 5 to 6 individuals and allowed to acclimate for 10 min before the start of training. Training was carried out for 30 min (TR1), whereby the snail receives a tactile stimulus (a poke) on the pneumostome each time it attempts to open it at the water’s surface^43^. This poke is sufficient to cause the pneumostome to close, but does not cause the snail to withdraw into its shell. To test for long-term memory (LTM) the snails received an identical procedure to the training session 24 h later.

Non-contingent (control): Training was identical to the contingent training above except that during training the control snail was poked in the vicinity of the pneumostome each time the snail with which it was paired received a poke, i.e. the control snail received an identical number of stimuli, but they were not contingent with pneumostome opening. During testing the control animal received contingent stimuli.

### Food aversion conditioning

Snails are trained to associate a recognised food resource that stimulates feeding behaviour (carrot) with a negative stimulus (exposure to KCl). Memory is demonstrated by a reduction in feeding behaviour in response to the carrot stimulus in trained animals.

Contingent (trained): Snails were food deprived for 48 h prior to training. They were placed individually into a small Petri dish (60 mm diameter) in 18 ml of artificial pond water and allowed to acclimate for 10 min. During the first session, 1 ml of artificial pond water was then added, followed 1 min later by a further 1 ml of pond water. The snails were then returned to their home aquaria. During the second session, 1 h following the first, snails were again acclimated to the small Petri dish in 18 ml of artificial pond water for 10 min. 1 ml of 5% carrot (w/v) water was then added and the bite rate (number of rasps) counted for 1 min. Following 1 min in carrot, 1 ml of 100 mM KCl was added and the snails were left in the resulting solution (0.25% carrot; 5 mM KCl) for a further 1 min. They were then removed and placed in their aquaria. To test for long-term memory 24 h later, snails were again placed in 18 ml of artificial pond water a small Petri dish and allowed to acclimate for 10 min. 1 ml of pond water was then added and the bite rate over 1 min counted, immediately followed by adding 1 ml 5% carrot solution and the bite rate counted for a further minute.

Non-contingent (control): To control for exposure to both carrot and KCl stimuli control training was carried out as above, except stimuli were presented non-contingently on the first day. Individual snails were placed in a small Petri dish (60 mm diameter) in 18 ml of artificial pond water and allowed to acclimate for 10 min. During the first session, 1 ml of artificial pond water was then added, followed 1 min later by a further 1 ml of 100 mM KCl and exposed for 1 min. The snails were then returned to their home aquaria. During the second session, 1 h following the first, snails were again acclimated to the small Petri dish in 18 ml of artificial pond water for 10 min. 1 ml of 5% carrot (w/v) water was then added and the bite rate (number of rasps) counted for 1 min. This was immediately followed by addition of 1 ml of artificial pond water; snails were left in the Petri dish for a further 1 min then returned to their aquaria. The memory test was identical to trained (contingent) animals above.

### Food appetitive conditioning

Snails are trained to associate a neutral stimulus that does not normally stimulate feeding behaviour (the odour of amyl acetate) with a food resource (exposure to sucrose solution). Memory is demonstrated by an increase in feeding behaviour in response to amyl acetate.

Contingent (trained): Snails were food deprived for 48 h prior to training. They were placed into a large Petri dish (140 mm diameter) in 90 ml of artificial pond water and allowed to acclimate for 10 min. During the first session, 5 ml of artificial pond water was then added, followed 2 min later by a further 5 ml of pond water and given a 2 min exposure period. The snails were then returned to their home aquaria. During the second session, 1 h following the first, snails were again acclimated to the large Petri dish in 90 ml of artificial pond water for 10 min. 5 ml of 0.08% amyl acetate water was then added and the bite rate (number of rasps) counted for 2 min. Following 2 min in amyl acetate solution alone, 5 ml of 13.4% sucrose solution was added and the snails were left in the resulting solution (0.004% amyl acetate; 0.67% sucrose) for a further 2 min. They were then removed and placed in their aquaria. To test for long-term memory 24 h later, snails were again placed in 90 ml of artificial pond water a large Petri dish and allowed to acclimate for 10 min. 5 ml of pond water was then added and the bite rate over 2 min counted, immediately followed by adding 5 ml 0.08% amyl acetate and the bite rate counted for a further 2 min.

Non-contingent (control): To control for exposure to both amyl acetate and sucrose stimuli, control training was carried out as above, except stimuli were presented non-contingently on the first day. Snails were placed in a large Petri dish (140 mm diameter) in 90 ml of artificial pond water and allowed to acclimate for 10 min. During the first session, 5 ml of artificial pond water was then added, followed 2 min later by a further 5 ml of 13.4% sucrose solution and the snails left for 2 min. The snails were then returned to their home aquaria. During the second session, 1 h following the first, snails were again acclimated to the large Petri dish in 90 ml of artificial pond water for 10 min. 0.08% amyl acetate water was then added and the bite rate counted for 2 min. This was immediately followed by addition of 5 ml of artificial pond water; snails were left in the Petri dish for a further 2 min then returned to their aquaria. The memory test was identical to trained (contingent) animals above.

### Data analysis – adult memory

Data analyses were carried out using SPSS 21 (SPSS Inc., Chicago, IL, USA). Adult memory performance at a population level was analysed using the change in behaviour between training and testing for each memory trait as follows: operant conditioning (breaths during memory test – breaths during training); aversive conditioning (bites during the memory test – bites during training); appetitive conditioning (bites during the memory test – bites during training). Data were analysed using ANOVA with training regime (contingent vs. non-contingent), order they experienced training regimes (4 levels) and origin (laboratory vs. ditch vs. river) as fixed factors, and population nested in origin as a random factor in the model, using the Satterthwaite approximation to estimate the degrees of freedom^46^. Student-Newman-Keuls pair-wise comparisons were used to carry out posthoc analyses.

To test for memory syndromes, all data on changes in behaviour between training and testing were converted so that they were on a positive scale, i.e. the greater the positive value the stronger the memory, and are presented in this format. Data were analysed using Pearson’s correlation.

### Training – juvenile memory

Operant conditioning was previously proposed to relate to threat avoidance behaviour in *L. stagnalis*^25^. To confirm whether memory following operant conditioning is indeed related to threat memory at a population level, cue association memory of predation threat in juvenile snails was tested using F2 juveniles from the same populations tested for adult memory traits. Juveniles were obtained by randomly selecting offspring from 50 F1 randomly selected adult snails (3-4 months old) per population that were not used to assess memory formation but retained as laboratory stock. Only river populations were used to assess this, as habitat type can significantly alter antipredator traits^4^. Juvenile *L. stagnalis* from river populations (R1-R4) were tested for memory of predation threat using methods adapted from Dalesman *et al*.^24^. Juvenile F2 snails (spire height 6 ± 0.5 mm) were pre-exposed to either control conditions or tench (*T. tinca*) plus alarm cue, their memory of predation threat was then tested 24 h following pre-exposure by exposing them during behavioural trials to either tench cues alone or control conditions. Tench cue was produced by holding three tench (10 ± 1 cm length) in 4 l of artificial pond water for 1 h; tench plus alarm cue was produced by crushing three juvenile snails (spire height 6 ± 0.5 mm) in 4 l of tench cue 24. Control water was artificial pond water alone.

Pre-exposure was carried out by placing 15 juvenile snails selected at random from the laboratory population into either 2 l of control water or tench plus alarm cue for 24 h. Water was fully aerated throughout, and snails were fed lettuce ad libitum during exposure. Following 24 h exposure to cue or control water, all snails were moved into new aquaria containing 2 l of control water for a further 24 h.

On the day of the behavioural trial, snails were randomly assigned to individual behavioural arenas 165 mm diameter x 60 mm depth (A.W.Gregory & Co. Ltd., U.K.) containing a central shelter, a longitudinally sectioned white PVC pipe, 36 mm long, 30 mm diameter, attached open side down to the centre using non-toxic sealant (Wickes Ultimate Sealant and Adhesive^©^, Wickes Building Supplies Ltd., U.K.) in 630 ml of control pond water and allowed to acclimate for 2 h. Following acclimation, either 70 ml of tench water or 70 ml of control water was added to each chamber in a randomised block design, such that an even number of snails were exposed to each of the pre-exposure conditions received either control or tench cue exposure during the behavioural trial. The position of each snail was recorded initially, and then every 5 min for 1 hour. Crawling above the water line is the primary antipredator response of juvenile *L. stagnalis*^4^,^24^,^47^, and so the proportion of time spent crawled out over the 1 h behavioural trail was used to assess antipredator behaviour.

Memory phenotype for each population was designated based on memory of adults snails in the F1 generation (see results Fig. 2): phenotype A: R1 and R2 (no evidence of memory following operant conditioning memory, but memory following food conditioning); and phenotype B: R3 and R4 (memory formation following operant conditioning but no evidence of food conditioning memory). Proportional data for time spent crawled out of the water were arcsine square-root transformed prior to analysis. Data were analysed using ANOVA with memory phenotype (A vs. B based on adult memory), pre-exposure conditions (control or tench plus alarm cue) and behavioural exposure conditions (control or tench cue) as fixed factors in the analysis, and population nested in phenotype as a random factor. Student-Newman-Keuls tests were used for posthoc pair-wise comparisons where overall significant effects were found.

## Additional Information

**How to cite this article**: Dalesman, S. *et al.* Habitat stability, predation risk and 'memory syndromes'. *Sci. Rep.* **5**, 10538; doi: 10.1038/srep10538 (2015).

## Figures and Tables

**Figure 1:**
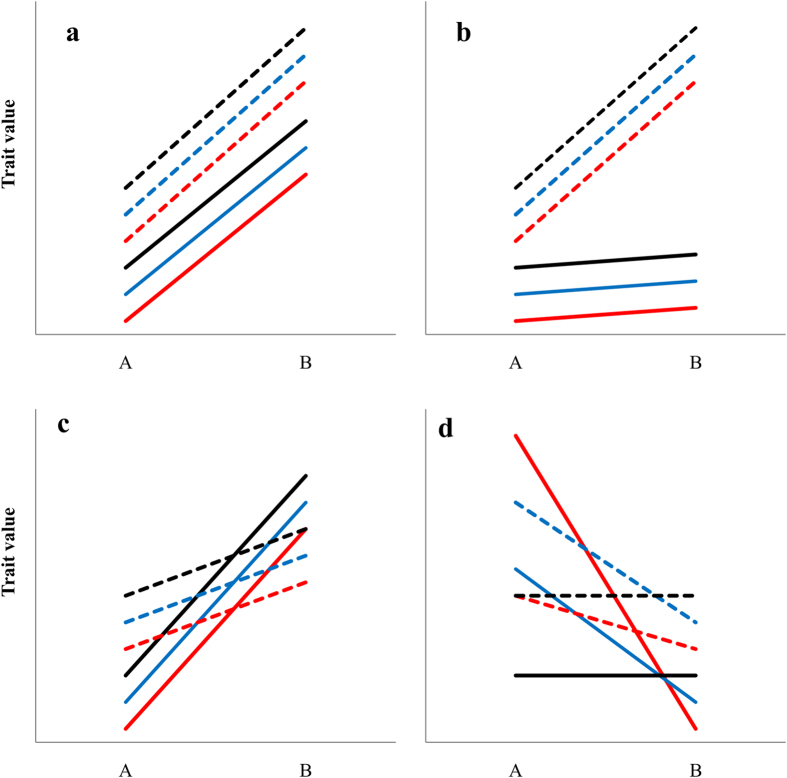
Demonstrating the relationship between behavioural syndromes (covariance between behavioural traits) and memory syndromes (covariance in plasticity across traits following memory formation) using two behavioural traits, e.g. antipredator behaviour and foraging behaviour (solid line and dotted line) in three individuals (red, blue and black). Arbitrary trait value (behaviour) is shown before and after memory formation. Panels demonstrate potential scenarios in which: **a**) memory formation is equal across behavioural traits and individual covariance between traits in maintained, demonstrating both a memory syndrome across traits and maintains the behavioural syndrome; **b**) memory only affects one trait, i.e. no memory syndrome across traits, but whilst it alters the mean difference between traits animals still demonstrate a behavioural syndrome following memory formation; **c**) all individuals demonstrate memory formation and the degree to which an individual alters its behaviour across traits is equal in within each trait (i.e. all individuals demonstrate a memory syndrome), however the behavioural syndrome is broken down; and **d**) no covariance among traits before memory formation and not all individuals demonstrate memory formation (i.e. no memory syndrome), however, after memory formation there is now significant covariance among behavioural traits (behavioural syndrome).

**Table 1:** Comparison of contingent training (trained) versus non-contingent training (control) within each individual population for each adult memory trait, showing the mean difference (trained - control), 95% confidence interval (CI) for the difference and effect size. * = significant difference found in posthoc pair-wise tests (SNK: P < 0.05).

**Source**	**Operant conditioning**		**Aversive conditioning**		**Appetitive conditioning**	
	**Mean difference (95% CI)**	**η^2^_p_**	**Mean difference (95% CI)**	**η^2^_p_**	**Mean difference (95% CI)**	**η^2^_p_**
Laboratory 1	-4.806* (-8.457,-1.155	0.165	0.452 (-3.615,4.518)	0.001	3.557 (-1.275,8.388)	0.058
Laboratory 2	-0.464 (-4.521,3.593)	0.002	-5.563* (-9.370,-1.755)	0.229	6.035* (0.781,11.288)	0.146
Ditch 1	-5.859* (-8.739,-2.979)	0.365	-2.247 (-4.959,0.465)	0.087	0.298 (-2.940,3.563)	0.001
Ditch 2	-0.220 (-3.919,3.479)	<0.001	-5.764* (-11.266,-0.262)	0.125	6.765* (0.741,12.789)	0.145
River 1	-2.380 (-5.469,0.708)	0.076	-5.653* (-8.862,-2.445)	0.251	9.528* (4.612,14.444)	0.283
River 2	-2.361 (-5.794,1.071)	0.058	-5.012* (-9.739,-0.286)	0.140	8.000* (3.525,12.475)	0.300
River 3	-4.737* (-8.069,-1.406)	0.197	-1.000 (-4.656,2.656)	0.009	-0.388 (-3.245,2.470)	0.002
River 4	-5.433* (-9.356,-1.511)	0.194	-0.196 (-4.591,4.198)	<0.001	1.333 (-0.257,2.942)	0.081
